# Two Sides of the Same Coin?—Treatment of Chronic Asthma in Children and Adults

**DOI:** 10.3389/fped.2019.00062

**Published:** 2019-03-11

**Authors:** Li Ping Chung, James Y. Paton

**Affiliations:** ^1^Department of Respiratory Medicine, Fiona Stanley Hospital, Perth, WA, Australia; ^2^School of Medicine, College of Medical, Veterinary, and Life Sciences, University of Glasgow, Glasgow, United Kingdom

**Keywords:** chronic asthma, pharmacotherapy, treatment, adult, children, guidelines

## Abstract

Globally, asthma is one of the most common chronic conditions that affect individuals of all ages. When poorly controlled, it negatively impacts patient's ability to enjoy life and work. At the population level, effective use of recommended strategies in children and adults can reduce symptom burden, improve quality of life and significantly reduce the risk of exacerbation, decline of lung function and asthma-related death. Inhaled corticosteroid as the initial maintenance therapy, ideally started within 2 years of symptom onset, is highly effective in both children and adults and across various degrees of asthma severity. If asthma is not controlled, the choice of subsequent add-on therapies differs between children and adults. Evidence supporting pharmacological approach to asthma management, especially for those with more severe disease, is more robust in adults compared to children. This is, in part, due to various challenges in the diagnosis of asthma, in the recruitment into clinical trials and in the lack of objective outcomes in children, especially those in the preschool age group. Nevertheless, where evidence is emerging for younger children, it seems to mirror the observations in adults. Clinicians need to develop strategies to implement guideline-based recommendations while taking into consideration individual variations in asthma clinical phenotypes, pathophysiology and treatment responses at different ages.

## Introduction

### Asthma Burden

Asthma is a chronic inflammatory disease of the airways with typical symptoms of wheezing, breathlessness and cough and variable airflow obstruction. The WHO estimates that some 235 million people currently suffer from asthma (1) and it is the commonest chronic disease of children worldwide. Asthma causes long term effects: it affects patient's quality of life, daily activities, their work and school attendance; it causes anxiety—to the patients themselves, and to their families and caregivers; it imposes a substantial economic burden on families and societies; and it causes death. Yet, with appropriate management most people with asthma can enjoy excellent symptom control and a good quality of life (1).

**Figure 1:**
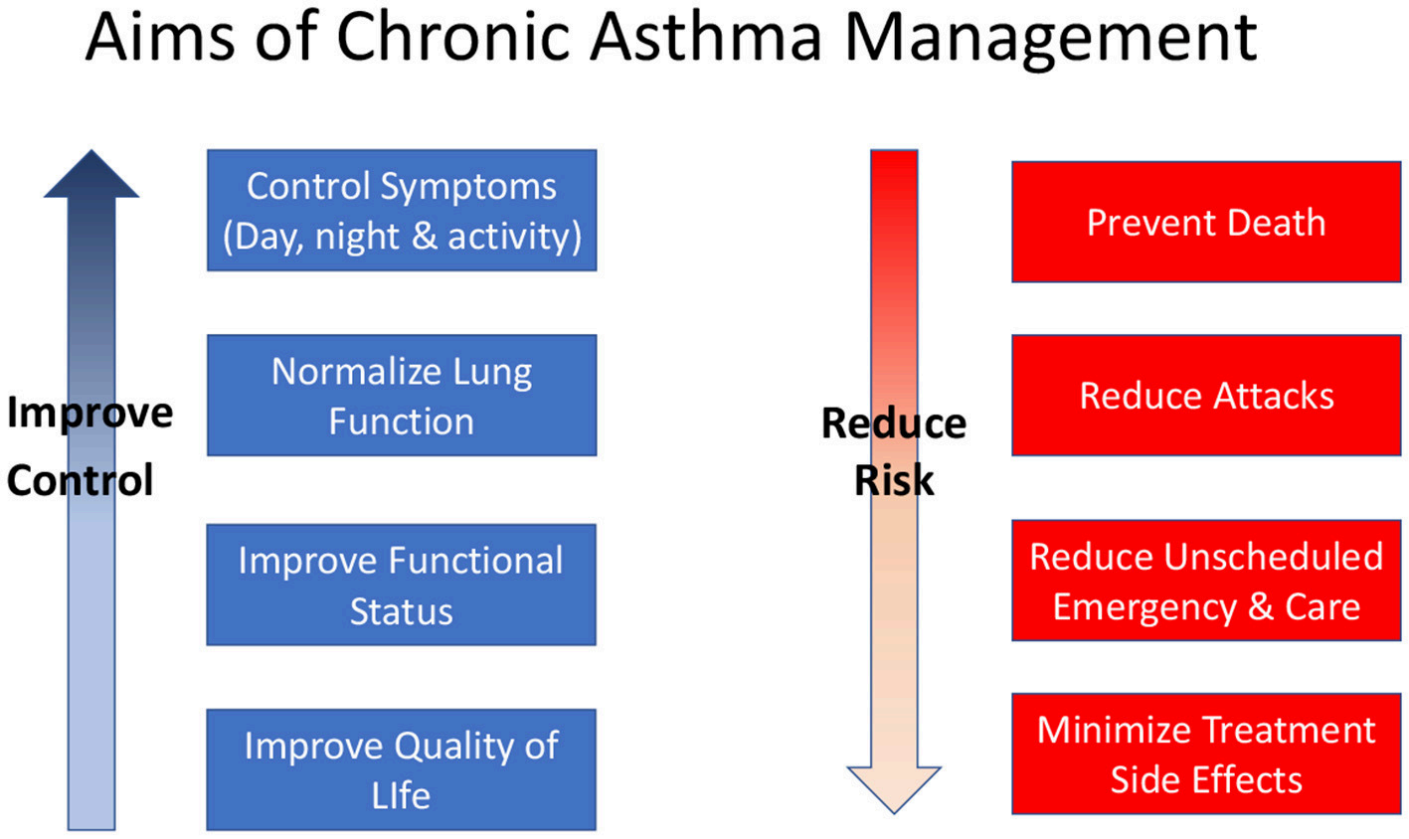
Aims of asthma management.

Control of asthma symptoms, reduction of future risk in terms of asthma attacks, and prevention of decline in lung function are recognized as the key goals of asthma management (Figure 1). These goals are relevant to all patients with asthma irrespective of age. Pharmacological therapies are the main tools for controlling asthma but non-pharmacological interventions such as allergen or irritant (especially tobacco smoke) avoidance, and education about the disease, inhaler device use and self-management strategies are also important.

### Asthma Phenotypes

There is increasing recognition of different clinical and patho-physiological patterns of asthma (‘phenotypes’) in both children and adults.

The most striking asthma phenotype is found in young children who present with wheezing attacks associated with viral infection, particularly rhinovirus infection, often with no symptoms between attacks. For many of these children, the wheezing attacks stop in later childhood (2). In older children, a more classic asthma pattern of attacks with chronic interval symptoms usually with an atopic background emerges (3). However, these patterns overlap and are unstable, at least in early childhood, and may change from one to another over times scales as short as a year (4, 5). Lung function testing in young children is generally not possible and making a diagnosis of asthma without objective testing has been criticized (6). The implications for the choice of treatments of the different phenotypes is complex and has resulted in a wide variety of approaches often compounded by a lack of evidence (7).

In adults, the pattern of airway inflammation is more diverse and the clinical presentation is often clouded by co-existing comorbidities especially in those with severe asthma (8, 9). Currently an emerging concept, but an important focus of asthma management in adults is the identification and management of pulmonary, extrapulmonary and behavioral “treatable traits”. The hope is that treatment of these modifiable elements (e.g., bronchodilator reversibility, eosinophilic airway inflammation, depression) even in the absence of a deeper understanding of the mechanisms of airway inflammation will improve patient outcomes (10). Although the concept of treatable traits has been developed in relation to adults it is also likely to be useful in children with asthma.

There are some patients, both adult and children, with “discordant” disease who suffer substantial symptom burden with little evidence of eosinophilic inflammation or those with high level of inflammation but few symptoms (11). However, the majority of patients will have asthma symptoms that parallel the degree of inflammation in which step-wise guideline recommended approach is likely to be effective.

This review summarizes evidence about differences in the approaches to asthma treatments in adults and children as currently recommended in commonly used asthma guidelines (12, 13) (Table 1).

**Table 1:** Comparison of recommended treatments for chronic asthma in adults and children from widely used international guidelines (12, 13).

**Asthma guideline treatment steps**	**Similarities in recommendations**	**Differences in recommendations, effectiveness or safety profile**
Step 1	• As needed SABA effective for all ages • Consider early use of ICS for adults and children ≥ 6 years old	
Step 2	• Regular low dose ICS for all ages	• Potential concern for effect of ICS on linear growth in children • Intermittent ICS may improve symptom control in adults and adolescents • Intermittent ICS in children <5 years with viral induced wheezing reduces the risk of severe asthma attacks.
Step 3	• Regular ICS recommended for all age groups • LTRA inferior to LABA or higher dose ICS in adults and children respectively • Allergen immunotherapy limited to carefully selected adults and children.	• Addition of LABA to low dose ICS as first preferred option in adolescents and adults • Moderate dose ICS preferred in children • Concern for LTRA-related neuropsychiatric effects in children and adolescents • ICS/formoterol as single maintenance and reliever therapy is another option for adolescents and adults; insufficient evidence for use in children <12 years old
Step 4	• ICS/LABA + SABA prn or ICS/fomoterol as single maintenance and reliever therapy recommended for adults and adolescents. •Higher dose ICS monotherapy provides little additional benefit in children and adults • Tiotropium is a safe, alternative add-on therapy for adults and adolescents; emerging supportive data for younger children • Theophylline no longer recommended for adults and children.	• Insufficient data for ICS/formoterol as single inhaler therapy use in children <12 years old • Specialist review recommended for children
Step 5	• Referral to specialist recommended for all ages • Review of medication adherence, comorbidities and risk factors important for all ages groups • Anti-IgE monoclonal therapy can be used for patients ≥ 6 years old • Anti-IL5 monoclonal therapy (mepolizumab) can be used in patients ≥18 years old and has been approved for use in children ≥ 6 years	• Use of anti-IL5 (mepolizumab) more extensively researched in adolescents and adults; use of younger children (≥ 6years old) approved based on extrapolated data. • Other advanced therapies such as macrolides and bronchial thermoplasty are recommended for adults; lacks data in children or adolescents.

*ICS, inhaled corticosteroid; LABA, long acting beta2 agonist; LTRA, leukotriene receptor antagonist; SABA, short acting beta2 agonist*.

## Guideline Recommended Approach for Chronic Asthma

### Step 1: As Needed SABA

For patients of all ages, as needed short acting beta2 agonist (SABA) reliever therapy is highly effective for rapid relief of asthma symptoms. SABA as the sole treatment is now only recommended for those with the mild and intermittent symptoms (<2 per week and no nocturnal awakenings), no history of asthma attacks in the previous year and normal lung function.

Historically, there has been a perception that patients with intermittent symptoms are not at risk of asthma attacks, a view increasingly challenged. Evidence from adult studies showed that airways inflammation occurs at a very early stage of the disease even in patients with intermittent asthma, albeit at a lower level than in those with persistent asthma (14, 15). Furthermore, early introduction of once daily inhaled corticosteroid (ICS) in adults and children > 5years with mild asthma symptoms reduced the risk of severe asthma exacerbation by almost half, with better asthma control although it did not affect lung function (16, 17).

This has led widely used international guidelines (12, 13) to update their recommendation for the consideration of ICS use as Step 1 treatment in all adults, adolescents and children over 6 years with mild asthma, even those with infrequent symptoms, to reduce the risk of severe, potentially life-threatening exacerbations (18–20).

### Step 2: Regular Low Dose ICS Plus as Needed SABA

#### Adults and Children (Age >5years)

Regular low dose ICS significantly improves lung function, quality of life and reduces asthma exacerbations in both adults and children with mild persistent asthma (13, 18). O'Byrne et al showed that introduction of low dose budesonide reduced the risk of asthma exacerbation by 60% and improved asthma control days by half (19). Selected studies in adults also showed less rescue SABA use (18). It was suggested that duration of therapy of at least 4 weeks may be an important determinant of overall treatment response (18).

The greatest benefits of ICS in adults and children older than 5 years old have been achieved when ICS therapy was started within 2 years of symptom onset (21, 22); this effectively halved the overall risk of severe or life-threatening exacerbations (16, 17). After this time, higher ICS doses are required for adults and there is a negative correlation between duration of symptoms and maximal increases in lung function (22–24). Low dose ICS appears protective against potential long-term decline in lung function following severe exacerbations. Interestingly, this effect is only seen in adults and children but not adolescents (25). It is unclear if this is due to the relatively small number of adolescents studied or relates to the improvements in asthma that have been noted during adolescence (26). Early initiation of ICS also improves asthma control and diminished subsequent needs for additional maintenance therapy (24).

One important difference in children compared with adults is a persistent concern about the impact of regular ICS in children on linear growth. As an example, in the START study Pauwels et al. reported that children aged 5–15years treated with budesonide showed reduced growth especially in the first year of treatment (16). However, other studies found that children attain normal projected adult height while on prolonged inhaled budesonide (21, 22).

The evidence on linear growth in children treated with ICS has recently been updated to take account of newer inhaled steroid molecules. The regular use of ICS at low or medium daily doses was found to be associated with statistically significant growth suppression during a 1-year treatment period in children with mild to moderate persistent asthma. The mean growth reduction was 0.48 cm/year in linear growth velocity and 0.61 cm change from baseline height during a one year treatment period. ICS-induced growth suppression appears to be maximal during the first year of treatment and less pronounced in subsequent years and appears to be more strongly associated with ICS molecule than with the device or dose in the low to medium dose range (27). A second review looking at dose response effects found a small but statistically significant group difference in growth velocity between low doses and low to medium doses, favoring the use of low-dose ICS (28). Thus the evidence confirms the relative safety of ICS in relation to growth effects but supports the use of the lowest effective dose of ICS, with regular monitoring of linear growth. It is surprising how many gaps still exist in the current evidence base about ICS and growth (29).

#### Preschool Children (Age <5years)

There is also strong and consistent evidence to support the use of daily ICS for preventing exacerbations in preschool children with recurrent wheeze, especially in those with persistent asthma symptoms (30). The effect of ICS appears to be stronger in those with a diagnosis of asthma compared with recurrent wheezing and is independent of age (pre-schoolers vs. infants), atopic condition, type of ICS, and mode of delivery (spacer vs. nebulizer) (31).

The evidence about growth suppression in pre-school children is limited. Nevertheless, because of concerns about side effects, an important question is whether ICS therapy can be targeted to those children most likely to respond? One recent study in children requiring step 2 treatment showed that while individual treatment responses were phenotypically diverse, children with aeroallergen sensitization and increased blood eosinophil counts responded best to a daily ICS as opposed to a leukotriene receptor antagonist (LTRA) or as needed-ICS. Daily ICS was associated with more asthma control days and fewer exacerbation (32). However, a very recent real-world study of large matched cohort analysis of anonymized UK medical record data found no evidence that stepping up therapy by adding ICS or monteleukast, compared with as-needed use of SABA, reduced wheezing/asthma attacks in a diverse population of preschool children with at least two documented prior wheezing episodes (33). In the absence of better tools to help target treatments, and in light of improved outcomes over time in preschool children a “wait-and-see approach” may be a clinically prudent approach for many with infrequent, intermittent attacks.

### Alternative Options

#### Low Dose Combination ICS/Long Acting Beta2 Agonist (LABA)

For steroid-naïve adults and adolescents with mild persistent asthma, ICS/LABA works faster in achieving GINA-defined good asthma control compared to ICS alone. However, there is little difference in exacerbation rate between the two therapies. In practice, the additional cost for ICS/LABA compared to low dose ICS may be an important consideration for the individual patient (34). Data as to whether there would be similar benefits in children is not available.

#### Intermittent ICS Regimes

Regular use of ICS raises concerns from patients and clinicians about adherence and steroid side effects. Poor adherence to ICS potentially results in patients relying on SABAs to control symptoms, possibly resulting in overuse.

Recently, it was shown that adults and adolescents with mild asthma using as-needed combination ICS and fast acting LABA had better asthma symptom control measured by an electronic diary compared to as-needed SABA (35). However, the magnitude of effect was inferior to regular low dose ICS therapy. A second trial in a similar cohort reported no difference in exacerbation rate when comparing ICS/formoterol as needed vs. regular ICS (36).

There is less evidence about the use of as needed ICS treatment in children. One study in children from 5 years upwards with mild well-controlled asthma stepping down from daily ICS found ICS and SABAs as needed more effective at reducing attacks than SABA use alone (37). Liner growth was 1.1 cm a year less when ICS was used regularly but not when used on an as needed basis.

In children under 5 years, the most common asthma phenotype is intermittent viral triggered wheezing attacks with no interval symptoms. In this group, intermittent ICS, usually as high-dose ICS at the first sign of an URTI for 7 to 10 days led to a 35% reduction in severe attacks (30). Intermittent ICS were associated with greater linear growth of 0.41 cm per year (i.e., less growth suppression) compared with daily treatment (38).

#### Oral Leukotriene Receptor Antagonist (LTRA) in Preschool Children

Because of concerns about ICS side effects and difficulties using inhaled therapies in young children, oral montelukast has been widely prescribed as an alternative to regular ICS. While early studies showed it to be effective, the most recent review found no evidence of benefit from the use of montelukast, continuously or intermittently on the number of wheezing episodes, unscheduled medical attendance, or oral corticosteroid use in preschool children with recurrent wheeze (39). There is also little evidence of benefit for other secondary outcomes (7).

### Step 3: One or two Maintenance Treatments Plus as Needed Reliever Therapy

For adults and adolescents with suboptimal symptom control or more than 1 exacerbation in the previous year despite treatment with low dose ICS, guidelines recommend low dose ICS/LABA as maintenance therapy plus SABA or ICS/formoterol as single inhaler therapy (12, 13). In children, at present, moderate dose ICS plus as needed SABA is the preferred treatment for children.

#### ICS/LABA Maintenance Plus SABA as Required

Historically, there were concerns that LABA may mask airway inflammation leading to potential adverse events including asthma exacerbations. To the contrary, the addition of LABA to ICS significantly increases the odds of achieving good overall asthma control as defined by guidelines (34). Combination low dose ICS/LABA therapy was highly effective at reducing the risk of exacerbations needing oral corticosteroid (19, 40, 41) and hospitalizations (40, 42, 43) in symptomatic adults and adolescents with mild to moderate asthma compared to ICS alone. The addition of LABA also leads to improvement in lung function (especially in those with lower baseline FEV_1_), proportion of symptom-free days (19, 44) and a slight reduction in rescue SABA use compared to same or higher dose of ICS (41, 43).

The advantages of adding a LABA have been less clear in children. In children with persistent asthma, the addition of LABA to ICS was not associated with a significant reduction in the rate of exacerbations requiring systemic steroids, but it was superior for improving lung function compared with the same or higher doses of ICS. There were no differences in adverse effects, with the exception of significantly lower linear growth over a year in the children treated with a higher ICS dose, with a mean difference of 1.21cm/yr. A trend toward an increased risk of hospital admission with LABA, irrespective of the dose of ICS, was noted as a matter for concern (45).

#### ICS/Formoterol as Single Maintenance and Reliever Therapy

Combination of ICS with formoterol as fast acting bronchodilator allows patients to use their regular maintenance inhaler also for rapid relief of symptoms. Single inhaler therapy is more effective at reducing mild to moderate exacerbations while providing similar levels of asthma control compared with medium dose ICS monotherapy or fixed dose of ICS/LABA and as required SABA (46–50). It has been suggested that using single inhaler for both maintenance and reliever therapy may improve adherence but definitive data is lacking.

The role of single maintenance and reliever therapy in children age 12 years and below is unclear at present and requires further study. It is not currently licensed for use in this way in this age group.

#### Alternative Treatment Options

##### Higher dose ICS

At the population level, further escalation to higher dose ICS use in adults and adolescents provides little added benefit but rather greater adrenal suppression (51, 52). Higher dose of ICS is inferior to combination ICS/LABA in reducing the risk of exacerbations requiring oral corticosteroids. Combination therapy is also superior in improving lung function, symptom control, and use of rescue SABA than a higher dose of ICS alone (44, 47).

In the past, written action plans commonly included advice about a temporary increase in inhaled steroid dose—usually doubling—in the early stages of an asthma exacerbation to reduce the severity of the attack and to prevent the need for oral steroids or hospital admission. The accumulated evidence suggested that this doubling approach was not effective (53).

However, the concept of intermittent ICS dose escalation to prevent asthma exacerbations has recently been revisited in two large studies. In a pragmatic and unblinded trial, McKeever et al. randomized adults and adolescents (*N* = 1922) on ICS maintenance therapy to receive a personalized management plan that included a temporary quadrupling of ICS when asthma control started to deteriorate compared to remaining on their usual ICS dose (54). They showed that a temporary quadrupling at the time of worsening asthma control resulted in a lower rate of severe exacerbations of asthma than no increase in the dose (45% vs. 52%).

In contrast, Jackson et al. studied children 5 to 11 years of age (*n* = 254) with mild to moderate asthma already on low dose ICS therapy and reported no difference in rates of exacerbation in those treated with quintupled dose of ICS compared with their usual ICS dose at the earliest sign of asthma deterioration (55).

The reasons for the differences in outcomes between these two studies are not completely clear. Differences in the size of study cohort and fewer than expected exacerbations in the pediatric study may be one reason and there may be differences in pathophysiology during exacerbations between adults and children. A more plausible explanation may lie in the fact the study by McKeever et al. was a pragmatic open label study with no monitoring of compliance. Increasing the ICS dose at the start of an exacerbation may have merely resulted in the patients starting a treatment they had been poorly compliant with before. In contrast, Jackson's study was a randomized control trial with electronic diary recording of treatment. Diary completion was > 70% during usual treatment and rose to around 98% of the days during the treatment (55, 56). Thus there may have been no headroom for a further impact of the increased ICS dose.

Given the bioequivalence dose of inhaled to oral corticosteroid and subsequent potential effects on adrenal suppression, it is also debatable whether the very high steroid dose used in the quadrupling or quintupling approach is necessarily better than a standard course of oral prednisolone (57).

##### Leukotriene receptor antagonists (LTRA)

Leukotriene receptor antagonists inhibit the pro-inflammatory effect of leukotrienes not completely suppressed by corticosteroid therapy. The addition of LTRA to symptomatic adult asthmatics already treated with regular ICS monotherapy leads to reduced exacerbations, better asthma control and better lung function (58, 59).

It is unclear if LTRA is superior to higher doses of ICS monotherapy (58) except in adults with aspirin-intolerant asthma. However, asthma symptoms, lung function, and rescue medication use are improved when LTRA is added to high doses of ICS (60). In this specific patient cohort, aspirin desensitization is also a safe, and effective option (61).

The addition of LTRA is less effective than adding LABA to ICS when one looks at comparative benefits in lung function, asthma symptoms, rescue medication use, and asthma related quality of life (62). The effect on oral corticosteroid-treated exacerbation was statistically superior with the addition of LABA than LTRA to ICS but with only an absolute difference of 2% between groups. However, this meta-analysis was dominated by 16 trials that included 6872 adults and adolescents; only 2 studies included 336 children (age 6–17 years). As such, it is unclear which adjunct therapy is best for children.

An earlier Cochrane 2013 analysis concluded there was no difference in asthma exacerbations needing oral corticosteroid or hospitalization comparing LTRA to same or higher dose ICS in children and adolescents with mild to moderate asthma (63). The paucity of randomized trials and heterogeneity in study design and reporting of published studies make it difficult to support its use as add-on therapy in children with moderate asthma (step 3 treatment).

In preschool children, there are as yet no trials comparing LTRA, or LABA, as an add on therapy to ICS.

Neuropsychiatric side effects have been reported as not uncommon in young children started on montelukast. One study (*n* = 106) found montelukast had been stopped in 16% of children because of neuropsychiatric effects (irritability, aggressiveness, and sleep disturbance), mostly within 2 weeks of starting therapy (64). Long term use in adults is not recommended unless there is clear symptomatic improvement after a trial of therapy (65).

##### Allergen immunotherapy

Allergen immunotherapy (AIT) is still the only-disease modifying treatment strategy for IgE-mediated allergic disease (66). Studies using both subcutaneous (SCIT) and sublingual treatments (SLIT) have shown some benefit in reducing asthma symptoms and bronchial hyper-reactivity in adults and children.

GINA guideline recommend SLIT for adults with rhinitis and allergy to house dust mite with exacerbations despite ICS, provided FEV_1_ is > 70% predicted (12). It reduces exacerbation rates in adults with moderate asthma but has minimal effect on asthma symptom control or quality of life (67). Its role in adults with compromised lung function requires further clarification. In a randomized control trial of subjects 14 years or older with mild to moderate asthma, those treated with sublingual house dust mite immunotherapy were able to reduce their ICS dose while maintaining asthma control (68).

In children, the most up to date evidence noted that there were no studies that evaluated asthma symptom using a validated tool and both study characteristics and outcomes were reported heterogeneously. There is moderate-strength evidence that SCIT may reduce long term asthma controller medication use in children with allergic asthma (68). Studies of SLIT have only studied children with mild/moderate asthma mono-sensitized to house dust mite. Local and systemic allergic reactions were common but anaphylaxis was reported rarely (69).

Current recommendations advise that allergen immunotherapy should not be given to patients with severe or uncontrolled asthma who are at increased risk for systemic reactions (70).

### Step 4: Two or More Maintenance Therapies Plus as Needed Reliever Medication

For adults and adolescents whose asthma is inadequately controlled on low dose ICS/LABA maintenance therapy, the two recommended step-up approaches are low dose ICS/formoterol single maintenance and reliever therapy or medium dose ICS/LABA as maintenance plus as needed SABA (44). In contrast, for children <12 years old because of concerns regarding medication side effects and the lack of sound evidence base, current guidelines recommend referral for specialist review (12).

*Post hoc* analysis of five large clinical trials suggests that ICS/formoterol as single maintenance and reliever inhaler may be the preferred option compared to guideline recommended treatment for various stages of asthma severity using low dose ICS monotherapy or same or higher fixed dose ICS/LABA regime (50). The magnitude of difference is more marked for those with more severe disease requiring GINA Step 4 treatment. Single maintenance and inhaler therapy prolongs the time to first exacerbation and reduces the overall risk of exacerbations at relatively low doses of ICS compared with higher fixed dose of ICS/LABA and as-needed SABA (46, 47, 50). The effect on hospitalization is less clear. Secondary outcomes such as lung function, rescue SABA use, symptom free days, and quality of life were also superior for single maintenance and reliever therapy than higher fixed dose ICS/LABA regime.

Again, the best option in children younger than 12 years old is unclear due to insufficient data.

#### Alternative and/or Additional Treatment Options

Several alternative options are available for adults and adolescents. However, robust data for their use in younger children is again currently lacking.

##### High dose ICS/LABA

While high dose ICS/LABA may be considered in adults and adolescents, the increase in ICS dose generally provides little additional benefit (34, 41, 71), and there is an increased risk of side effects, including adrenal suppression (72). A high dose is recommended only on a trial basis for up to 6 months when good asthma control cannot be achieved with medium dose ICS/LABA and /or a third controller (12).

##### Long acting muscarinic antagonist (LAMA)

The long-acting anticholinergic tiotropium, delivered once daily via a mist inhaler, is approved for the treatment of asthma in the EU and the USA with the license recently extended to include children with severe asthma over 6 years of age. The GINA guidelines currently position tiotropium as an add on therapy option at step 4 in patients aged ≥12years with a history of exacerbations (12).

For adults treated with ICS, LAMA prescribed as add on therapy has been shown to reduce the risk of exacerbations and improve lung function but made no difference in quality of life (73). In a study of 210 moderate asthmatics, tiotropium bromide added to ICS offered marginal improvements in peak flow and symptoms compared to doubling dose of ICS. The effect was considered non-inferior to salmeterol. However, there was the possibility of carry-over effects due to the cross-over design (74, 75). Cochrane analysis of 3 trials (76–78) comparing addition of tiotropium mist inhaler in adults with severe asthma already treated with ICS/LABA combination therapy showed definitive improvement in trough FEV_1_ and fewer exacerbations (79).

In children over 6 years with symptomatic moderate or severe asthma, tiotropium treatment led to improvement in lung function and asthma control (80), similar to that found in adult studies. However, the data in children is less extensive and generally short term with the longest study lasting 48 weeks (81). In younger children, one small 12 week RCT of children aged 1–5years study showed tiotropium was safe, led to a reduction in asthma attacks but did not improve daily asthma symptom scores. These findings need to be confirmed in larger studies (82).

Both in adults and children tiotropium has a safety profile comparable to placebo.

##### Theophylline

The addition of slow release theophylline to low dose budesonide for moderate asthma is just as effective as high dose budesonide in improving lung function and reducing symptoms and rescue SABA use in adults but has little effect on exacerbation rate over 3 months duration (83). Theophylline is a less effective bronchodilator than beta2 agonists and associated with high risk of adverse effects.

In the past, oral xanthines were used as a first line preventer treatment (at Step 2) for children with asthma. Although there is weak evidence that theophyllines were better than placebo, they are no longer used because ICS were shown to be more effective at improving symptoms and reducing asthma attacks (84). When low dose ICS fail to control asthma, oral theophyllines have been used as one of the available add-on options and there is weak evidence that adding theophylline to ICS treatment improves symptom control and reduces exacerbations (85). However, current recommendations are that theophyllines should only be tried when the addition of LABA and LTRA have both failed.

Theophyllines have adverse effects including headaches and nausea if therapeutic concentrations are exceeded. There are also significant interactions with some commonly used drugs which inhibit theophylline clearance e.g., erythromycin which can result in increased theophylline levels and resulting toxicity.

### Step 5: Refer for Expert Review and Add-On Therapy

The great majority of adults with severe asthma not responding to high dose treatments are “difficult-to-treat” because of comorbidities and risk factors that mimic or worsen asthma control (86, 87) and the management of these other factors is as important as the asthma treatments in improving the outcomes of patients with severe asthma. The situation is similar in children where only the minority will have genuinely therapy resistant asthma (88).

Poor adherence with treatment is a particularly important cause of difficult to treat asthma in both adults and children. Improving adherence is difficult. A variety of interventions have been shown to lead to improvements including adherence education, the use of electronic trackers or reminders, simplified drug regimens and school-based directly observed therapy. However, because of uncertain and inconsistent impact on clinical outcomes such as quality of life and asthma control the clinical relevance of the improvements has been less clear with many studies affected by concerns about risk of bias and inconsistency (89). The most recent studies in adults and children have shown that a combination of electronic monitoring and biofeedback can improve adherence and asthma outcomes (90–92). In one adult study, a programme of adherence and inhaler technique assessment resulted in only 27% remaining refractory to their asthma treatment (92). Hence, such approaches may offer a more effective approach in the future.

A number of add-on options are available for those with severe therapy resistant asthma. But prior to the use of these therapies, it is important to follow a systematic approach in the assessment and management of “difficult-to-treat” severe asthma—to confirm diagnosis, address poor adherence and to identify and manage comorbidities and risk factors (93, 94).

The following add-on options are best reserved for well-phenotyped patients with “severe treatment-refractory” asthma. The bulk of the evidence for their use at present is in adults and adolescents.

#### Anti-IgE Monoclonal Therapy

Omalizumab can be used for adults and children over 6 years of age with inadequately controlled severe allergic asthma despite optimized therapies (12, 13). The published data mostly related to adolescents and adults with only 3 randomized studies exclusive to children or adolescents (95–97). Most of the published data included subjects with moderate to severe asthma where omalizumab appears equally effective across different ages at reducing the risk of exacerbations and hospitalizations (98–100). Benefits are also seen in asthma symptom control for adults and children whilst other outcomes such as quality of life and rescue medication use have mainly been assessed in adults with modest improvements.

In addition, omalizumab allows for reduction or withdrawal of inhaled corticosteroid use but the latter option must be considered with caution (98). In children with severe disease omalizumab can reduce the burden of corticosteroids (101) and might be an effective alternative to oral corticosteroids (OCS) although a large direct OCS-sparing trial in children is required to confirm this.

In clinical use in both adults and children omalizumab has proved safe with few serious adverse effects (98).

#### Anti-interleukin 5 (IL-5) Monoclonal Therapy

Newer therapies targeted at IL-5 (mepolizumab, reslizumab) or the IL-5 receptor (benralizumab) are effective for adolescents and adults with severe eosinophilic asthma at high risk of exacerbations or with high symptom burden despite high dose ICS and another maintenance therapy (102). The effectiveness of subcutaneous mepolizumab (103, 104) and benralizumab (105, 106) in reducing exacerbations and hospitalizations is well-documented. They also improve asthma control, quality of life with modest gain in lung function and reduce the need for oral corticosteroid (106–108). Similar benefits were demonstrated with reslizumab only when administered intravenously which may impact its use in clinical practice (109, 110).

Randomized trials for all three anti-IL-5 therapies recruited patients over 12 years and included a few adolescents; studies using mepolizumab in children age between 6 and 11yr are underway. The EMA has recently approved mepolizumab for use in children 6–17years but this is based on an extrapolation of data from the efficacy and safety data from the Phase III studies in the mepolizumab severe asthma development programme for patients 12 and over.

#### Possible Alternatives for Adults Not Included in Guidelines: Macrolides, Bronchial Thermoplasty

##### Macrolides

Macrolides have antibacterial, antiviral and immunomodulatory effects and are shown to be effective in different asthma phenotypes (111). Adults with persistent asthma despite two or more maintenance therapies including ICS have fewer exacerbations and better quality of life when treated with azithromycin for 6-12months (111, 112). Macrolides are well–tolerated but microbial resistance is a potential concern with long term use. To date, there is no reported increase in infections related to macrolide use.

At present, there is no evidence that regular treatment with macrolides improves asthma control in children. Intermittent use of azithromycin in preschool children at the time of asthma attacks has been investigated. One study found that Azithromycin shortened the duration of episodes of asthma-like symptoms in young children aged 1–3years (113); in a second study in preschool children presenting to an emergency department, 5 days azithromycin (vs. placebo) neither reduced the duration of respiratory symptoms nor time to respiratory exacerbation in the following 6 months after treatment (114).

##### Bronchial thermoplasty

Bronchial thermoplasty is a non-pharmacological, endoscopic treatment for subjects aged ≥18 years with severe persistent asthma that is not well-controlled with ICS and LABA. Bronchial thermoplasty provides lower rates of exacerbations and modest improvements in quality of life but no difference in asthma control scores in patients with moderate to severe asthma (115–117). The procedure may be associated with temporary deterioration in asthma control needing hospitalization but is not associated with significant adverse respiratory effects on follow up out with the treatment phase. Its role in adult patients with severe asthma remains unclear especially in those with poor lung function. Based on current data, bronchial thermoplasty is not recommended by guidelines and it should only be used in carefully considered patients in the setting of a registry to allow independent data collection and in centers experienced in the technique. At present, there is no data on the use of bronchial thermoplasty in young people with severe asthma <18years of age.

##### Chromones (nedocromial sodium and sodium cromoglycate)

Chromones have favorable safety profile but low clinical efficacy compared to ICS in adults and children (118). The most recent Cochrane systematic review of studies in children judged there was insufficient evidence to be sure about the efficacy of sodium chromoglycate over placebo and was concerned that publication bias is likely to have overestimated the beneficial effects of sodium chromoglycate as maintenance therapy in childhood asthma in the past. They may have some role in those with exercise induced bronchospasm who are unresponsive to SABA pre-exercise and regular ICS for underlying asthma, as well as those intolerant of bronchodilators (119).

### Approach to Adjusting Maintenance Treatment

Pharmacological management of asthma is based on continuous cycle of assessment, treatment and review to allow up or down titration of maintenance therapies to maximize patient outcomes using minimal treatment at various stage of the disease (12, 13).

#### Control Based Treatment Adjustment

Most guidelines recommend a change in management based on measures of symptom control with or without other risk factors such as compromised lung function or a history of exacerbations. Symptom based approaches are essentially all that is available in young children because of the fact they cannot co-operate with standard lung function tests.

For many patients in primary care, symptom control is a good guide to reduced risk of exacerbations. However, it is important to be mindful that in some patients, there may be discordance between responses in symptom control and asthma attacks. In preschool children, particularly, many children will have severe attacks of wheezing but no symptoms in between attacks.

#### Alternative Strategies for Adjusting Asthma Treatment Based on Eosinophilic Markers (Sputum Eosinophil Count or Exhaled Nitric Oxide)

Airways inflammation in asthma can be predominantly eosinophilic or non-eosinophilic. While ICS are the major preventer treatment to control symptoms, ICS are more effective in reducing symptoms in patients with eosinophilic inflammation than in those with neutrophilic inflammation (120). There has therefore been interest in whether tailoring asthma treatment based on objective eosinophilic inflammation improved asthma outcomes. Two approaches have been used to date: examination of eosinophil counts in induced sputum samples; or the measurement of exhaled nitric oxide.

The most up-to-date synthesis of the evidence concluded that children and adults randomized to either eosinophilic marker strategy were significantly less likely to experience an exacerbation during the follow-up period (4.5–24months). The exacerbation rate was also lower in adults with either strategy compared to controls, but not in children. For both strategies in adults and children, there was no difference for all secondary outcomes (FEV_1_, asthma control test score, asthma quality of life, beta agonist use). There was also no difference in the final ICS use in either adults or children for either strategy (121).

Exacerbations are one, albeit important, asthma outcome; other outcomes such as symptom control and lung function also need consideration. Why there is discrepancy between exacerbations and other asthma outcomes is not understood but it has been noted in other studies involving the newer monoclonal drugs targeted at eosinophilic allergic pathways such as mepolizumab (103).

Currently, sputum induction is restricted to laboratories and clinics with specific expertise. It is technically demanding and time consuming and not always successful, particularly in younger children. Universal use of FeNO would be a substantial extra cost if used for all asthma patients and there is a yet no evidence-based algorithm on how to adjust treatment based on FeNO levels. Such an approach is most likely to benefit those with frequent asthma exacerbations.

In studies limited to non-smoking adults, FeNO >50ppb was predictive of good short-term response to ICS (122). However, there are no studies examining the long-term safety with regard to exacerbations or withholding ICS in patients with low initial FeNO.

### Asthma Treatment at Different Ages – Challenges to Asthma Guidelines

The evidence base for the management of children with asthma, particularly young children, is often quite limited and has been compounded by a number of problems. Children under the age of 5–6years are usually not able to co-operate with lung function test and as result objective outcomes are commonly not available. Trials of treatment are more problematic without objective outcomes. New drugs are usually not tested in children until they have been extensively tested in adult patients. Recruiting and retaining children and their parents into randomized studies is difficult and as a result trials are often more limited in scale and duration. The consequence is that the evidence base for asthma treatments is often much smaller than available for adults. However, when an evidence base does emerge it is surprising how often it mirrors the that from adult studies. The value of ICS in preventing asthma exacerbations in young children would be an obvious example.

Asthma Guidelines summarize “population level” evidence and provide broad and generalized recommendations about asthma management (12, 13). However, it is now accepted that asthma is a heterogeneous disease and adjusting asthma treatment that takes account of differing phenotypes is one of the most important current challenges.

Despite publication of many evidence-based guidelines and the availability of effective therapies, there is widespread concern that asthma control in adults and children often remains poor and that asthma attacks and even deaths from asthma are not improving (123, 124). This is largely because there is a need to take account of “patient level” factors and tailor treatments to individuals. Such factors include any individual characteristics, preferences, risk factors, comorbidities or phenotype that predict or influence a patient's likely response to treatment, together with practical issues such as ability to use a particular inhaler, adherence, and affordability. Finally, patients with asthma, including children and their parents, often have different treatment goals from their physicians and want to balance the aims of optimizing asthma management against the side effects or inconvenience of taking regular medication necessary to achieve asthma control (13).

## Conclusion

Asthma can significantly impact on all facets of life for patients across all age groups. Effective management strategies are broadly summarized by local and international guidelines. Overall, the evidence for pharmacological approach are more extensive and more robust for adults than that for younger children. For milder disease, the use of ICS as the initial maintenance therapy and SABA as needed appears universally effective for children, adolescents and adults. However, the preferred choice of subsequent add-on therapies differs and the evidence base for advanced therapeutic options is mostly based on studies in adolescents and adults. Clinicians are currently challenged with the need to develop management strategies that best caters for individual differences in asthma presentation and management. Accounting for differences in pathophysiological mechanisms, asthma phenotypes and treatment responses at different ages remains one of the most significant of these challenges.

## Author Contributions

Both authors contributed to the conception and preparation of this manuscript. LC contributed to the first draft and the content relating to asthma management in adults. JP provided his summary and expertise on treatment of asthma in children. Both authors contributed to manuscript revisions, read and approved the submitted version.

### Conflict of Interest Statement

LC has received honorarium for lectures and advisory board meetings from GlaxoSmithKlein, AstraZeneca, Boehringer Ingelheim, Menarini, and Novartis.

The remaining author declares that the research was conducted in the absence of any commercial or financial relationships that could be construed as a potential conflict of interest.
